# Altered juvenile fish communities associated with invasive *Halophila stipulacea* seagrass habitats in the U.S. Virgin Islands

**DOI:** 10.1371/journal.pone.0188386

**Published:** 2017-11-21

**Authors:** Lauren K. Olinger, Sarah L. Heidmann, Allie N. Durdall, Colin Howe, Tanya Ramseyer, Sara G. Thomas, Danielle N. Lasseigne, Elizabeth J. Brown, John S. Cassell, Michele M. Donihe, Mareike D. Duffing Romero, Mara A. Duke, Damon Green, Paul Hillbrand, Kristin R. Wilson Grimes, Richard S. Nemeth, Tyler B. Smith, Marilyn Brandt

**Affiliations:** Center for Marine and Environmental Studies, University of the Virgin Islands, St. Thomas, United States Virgin Islands, United States of America; Leibniz Centre for Tropical Marine Research, GERMANY

## Abstract

Caribbean seagrass habitats provide food and protection for reef-associated juvenile fish. The invasive seagrass *Halophila stipulacea* is rapidly altering these seascapes. Since its arrival in the Caribbean in 2002, *H*. *stipulacea* has colonized and displaced native seagrasses, but the function of this invasive seagrass as a juvenile fish habitat remains unknown. To compare diversity, community structure, and abundance of juvenile fish between *H*. *stipulacea* and native seagrass beds, fish traps were deployed in four nearshore bays around St. Thomas, U.S. Virgin Islands. Traps were deployed in Frenchman, Lindbergh, and Sprat Bays for 24 h intervals in patches of bare sand, patches of *H*. *stipulacea* and patches of the native Caribbean seagrasses *Thalassia testudinum* and S*yringodium filiforme*. Traps were then deployed in Brewers Bay for 12 h intervals in stands of *H*. *stipulacea* and *S*. *filiforme*. Relative and total abundances of juvenile fish, identified at least to family, were compared across treatment habitats for each trap deployment period. The catch from *H*. *stipulacea*, compared to native seagrasses, comprised a greater abundance of nocturnal carnivores *Lutjanus synagris* (family Lutjanidae) and *Haemulon flavolineatum* (family Haemulidae). Additionally, the herbivore species *Sparisoma aurofrenatum* (family Labridae) and *Acanthurus bahianus* (family Acanthuridae) and the diurnal carnivore species *Pseudopeneus maculatus* (family Mullidae) were relatively scarce in *H*. *stipulacea*. The catch from sand was much smaller, compared to vegetated habitats, and comprised only *L*. *synagris*, *H*. *flavolineatum*, and *H*. *aurolineatum*. These results provide evidence of reduced family diversity and altered juvenile fish assemblages in *H*. *stipulacea*, driven by an abundance of some nocturnal carnivores and scarcity of herbivores and diurnal carnivores. The findings from the present work underpin the need for further investigation and mitigation of this invasion, particularly where *H*. *stipulacea* is driving seascape-alterations of key juvenile fish habitats.

## Introduction

Nearshore Caribbean seagrasses are essential habitats, especially for juvenile fish that depend on these shallow, vegetated seascapes for shelter and food resources [1–3]. Seagrasses are morphologically diverse and host unique epibiota communities. Consequently, seagrass composition governs the degree of protection and food resources provided by the habitat and thus the juvenile assemblages associated with it. A seascape comprising a patchwork of different seagrasses will, therefore, provide suitable habitats for a diversity of juvenile fish [4, 5]. Juvenile assemblages are further influenced by the spatial arrangement of seagrasses in relation to coral reefs and oceanic currents [6, 7]. The juveniles of some reef-associated fish are more abundant in seagrasses that are closer to reefs [3], and larval delivery to settlement habitats is regulated by oceanic circulation [8]. In addition, considerable changes in juvenile assemblages occur over time. Seasonal variation is driven by fluctuating water temperatures, rainfall, and wind speed and direction [9, 10], and prominent diel variation is driven by crepuscular migrations between seagrasses and nearby coral reefs [2, 11, 12].

Life-stage and species-specific habitat preferences play a complex role in structuring juvenile fish assemblages within seagrass habitats. The juveniles of some species initially inhabit sheltered back-reef seagrass meadows, gradually expand their range as they grow, and eventually undergo ontogenetic migrations to coral reefs [3, 13]. Range expansion can result from age-specific dietary changes, and ontogenetic migrations may be triggered also by dietary changeover or by sexual maturation, depending on species [14]. Further specificity in habitat preference is driven by species-specific feeding preferences, diel activity, and mobility [7,15]. A need for food may drive juveniles to favor one seagrass foraging habitat over another during waking hours, and this preference is based on nutritional content of seagrass blades or associated epibiota [16]. Conversely, a need for shelter may drive juveniles to migrate to protective coral reefs or structurally complex seagrass beds during resting hours [17,18]. Transient groups that migrate between seagrasses and reefs in constant pursuit of adequate shelter or foraging habitat require many suitable habitats within their range [19]. Alteration of seagrass ecosystems can cause reduction of preferred juvenile habitats, spatial isolation of seagrasses and coral reefs, and ultimately a loss of ecologically- and commercially-important coral reef fish [20].

The invasive seagrass *Halophila stipulacea*, native to the Red Sea and the Western Indian Ocean, was first observed in the Caribbean on the island of Grenada in 2002 [21]. *H*. *stipulacea* has since expanded to nineteen other Caribbean islands, often at the expense of native seagrasses [21, 22, 23]. On the west coast of Dominica, *H*. *stipulacea* spread at an alarming rate, expanding to cover over 600 ha in five years and displacing native seagrasses *Halodule wrightii*, *Syringodium filiforme*, and *H*. *decipiens* [24]. *H*. *stipulacea* was first observed in the United States Virgin Islands (USVI) on the island of St. John in 2012 [23], and in 2013 it was reported on the neighboring island of St. Thomas [25]. In September 2016, fragments of *H*. *stipulacea* were found on St. Croix, indicating that this seagrass may soon be established around all three major islands of the USVI [26].

The success of *H*. *stipulacea* is a result of this species’ colonizing ability and resilience; it can rapidly invade newly-cleared seabeds after blowouts, withstand frequent disturbances similar to climax species, and rapidly recolonize or spread to new areas via fragmentation [22, 24]. This invasion may also be facilitated by increasingly poor environmental conditions in many nearshore Caribbean ecosystems. Previous findings suggest that dense mats of *H*. *stipulacea* grow exclusively in environments with high nutrient concentrations, and this dense growth form is more effective than the sparse growth form at displacing native seagrasses [27]. Poorly flushed Caribbean embayments that are subjected to high nutrient inputs may be at greater risk of takeover by densely matted *H*. *stipulacea* and loss of native seagrasses.

The invasion of *H*. *stipulacea* in the USVI and wider Caribbean often corresponds to displacement of native seagrasses, but the impact of this invasion on the juvenile fish that rely on these affected habitats is poorly understood [28]. Alteration of juvenile fish assemblages have occurred elsewhere as a result of invasion by habitat-forming species such as the common reed *Phragmites australis* [29], and the green alga *Caulerpa taxifolia* [30]. One recent study conducted in Dominica found that *H*. *stipulacea* supported greater abundances of epibiont food resources and larger overall fish (inclusive of all life stages), compared to the native seagrass *S*. *filiforme*. Fish abundance, however, did not differ between the native and invasive seagrass; it was cautioned that this could be due to low experimental power (27% power to detect a 50% difference at alpha = .05). Interestingly, juveniles were twice as abundant in *S*. *filiforme* compared to *H*. *stipulacea*, suggesting that *S*. *filiforme* habitats in Dominica may be a better juvenile habitat [22].

The present study investigated how juvenile fish diversity, community structure, and abundance differ between habitats composed of *H*. *stipulacea*, native seagrass *S*. *filiforme*, native seagrass *Thalassia testudinum*, and sand in four shallow bays of St. Thomas, USVI. The exclusive focus on juveniles in the present work is because, aside from the value of seagrasses to juvenile fish, little is known about seagrass-associated juvenile assemblages in St. Thomas; most research has concerned reef-associated adult populations around St. Thomas (e.g., [31, 32]). To explore broad-scale patterns in habitat use, fish traps were deployed at three sites for 24 h periods within patches of *H*. *stipulacea*, *S*. *filiforme*, *T*. *testudinum*, and sand. To investigate diel variation in habitat use, traps were next deployed at a fourth site for 12 h periods within patches of *H*. *stipulacea* and *S*. *filiforme*.

The first hypothesis in this study was that there would be less diverse and less abundant juvenile fish associated with *H*. stipulacea compared to native seagrass species, as the invasive may not provide adequate shelter or food resources [28, 33]. The second hypothesis was that there would be more diverse and abundant juvenile carnivores in *H*. *stipulacea*, due to recent findings that *H*. *stipulacea* supports abundant carnivore food resources [22]. The third hypothesis in the present study was that there would be less diverse and less abundant juvenile herbivores in *H*. *stipulacea*, as the invasive likely does not provide adequate shelter and may be associated with fewer or suboptimal herbivore food resources. Though not studied in the Caribbean, Mediterranean *H*. *stipulacea* was associated with less diverse and abundant algal epiphytes compared to other seagrasses [34]. Hypotheses were tested equally during 24 h and 12 h deployments.

## Methods and materials

### Ethics statement

This study was carried out in strict accordance with the American Fisheries Society Guidelines for the Use of Fishes in Research. A passive capture method was used, specimens were handled briefly, and no invasive techniques were used. This protocol was approved by the University of the Virgin Islands IACUC (IRB #747807–1). No endangered or protected species were involved in this study. All study sites are publicly-owned waterways, and fish capture was conducted with permission from the Department of Planning and Natural Resources Division of Fish and Wildlife (permit #DFW15053T).

### Site selection

**Fig 1:**
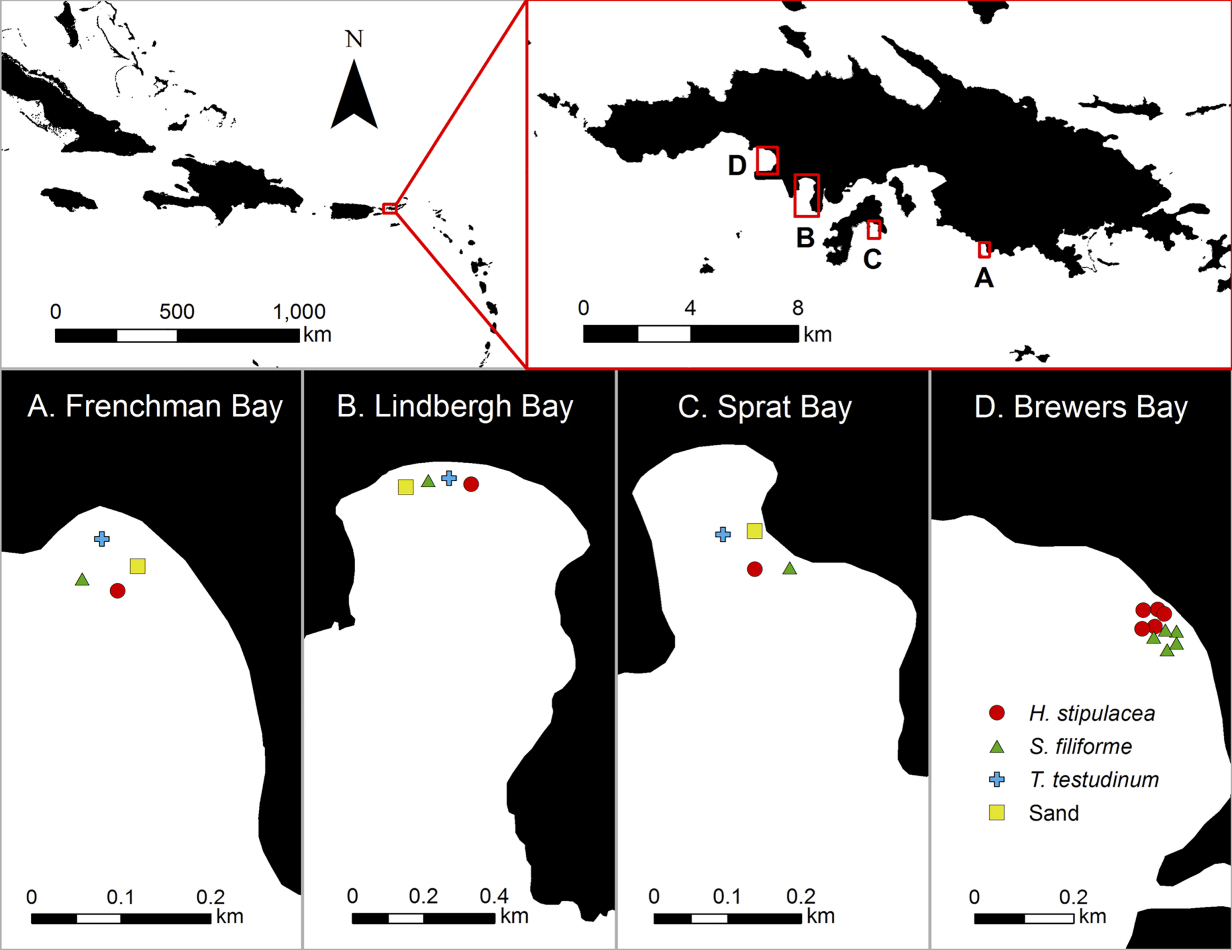
Study sites in St. Thomas, United States Virgin Islands. (A) Frenchman Bay (18°18'51.11"N, 64°54'27.52"W). (B) Lindbergh Bay (18°20'6.13"N, 64°58'6.93"W). (C) Sprat Bay (18°19'14.05"N, 64°56'39.98"W). (D) Brewers Bay (18°20'37.26"N, 64°58'40.59"W).

Data collection for this study occurred during February 2016 across four sites located along the south side of St. Thomas, USVI (Fig 1). Frenchman, Lindbergh, and Sprat Bays were selected for 24 h trap deployment sites (Fig 1A–1C) because each bay contained distinct monotypic stands of *H*. *stipulacea*, *S*. *filiforme*, and *T*. *testudinum*. Brewers Bay (Fig 1D) was selected for 12 h trap deployments because it contained particularly large monotypic stands of *H*. *stipulacea* and *S*. *filiforme*.

The calmest conditions for southern St. Thomas bays occur during winter months (December to March) because they are sheltered on the leeward side of the island from seasonal northeast winds. Between April and November, prevailing winds are out of the southeast and expose easternmost bays (Frenchman and Sprat) to high winds and wave action. Westernmost bays (Brewers and Lindbergh) are the calmest year-round due to their leeward orientation.

At every site, prospective seagrass patches were identified, and their size, depth, distance from shore, and distance from other patches were noted. Habitat characteristics were then surveyed in each prospective patch. Five 0.25m^2^ quadrats were placed randomly in each patch, and percent cover of the target seagrass species in that quadrat was estimated. Then the canopy height was recorded by measuring the length of one shoot on the bottom right of the quadrat.

Only monotypic stands were chosen as treatment habitats. Monotypic stands were defined in this study as patches that were larger than 10 m^2^ with a greater than 75% cover of the target seagrass species. Additionally, only patches at similar depths (2–4 m), close to shore (<100 m), and relatively isolated from other seagrass species (>10 m) were chosen as trap deployment locations. Sand habitats with no structure or vegetation over at least a 10 m^2^ area were also identified to serve as treatment habitats for 24 h trap deployments. A small threshold area (10m^2^) could have led to the detection of edge effects, in which fish community structure is influenced by migration, predation, and competition at seagrass-sand and seagrass-reef interfaces [35, 36]. To minimize edge effects, seagrass or sand patches in the visible range (within ~25 m) of well-developed aggregate reef were excluded from this study, and traps were deployed in the center of each patch.

### Experimental methods

Fish were collected using unbaited traps (53 x 53 x 53 cm) with 1 cm square mesh made from vinyl-coated wire. Traps had a double funnel opening and resembled rectangular Antillean fish traps. Traps were used because they non-selectively target juvenile fish and do not affect fish behavior, unlike other capture techniques such as hook and line fishing or trawling [37, 38]. Some drawbacks to using traps include risks of conspecific attraction [39], high variation across traps [40], repeatedly catching the same individuals [38], and predation [41]. Traps permitted capture of medium and large juveniles, but the 1 cm mesh precluded capture of the smallest juveniles. Exclusion of the smallest juveniles was determined to be permissible. It has been argued that, because mortality is high among the smallest juveniles, it is more reliable to characterize assemblages of medium and large juveniles that have spent more time in their nursery habitat [42].

To investigate broad-scale patterns in juvenile assemblages across habitats and sites, 24 h trap deployments were conducted at three sites (Frenchman, Lindbergh, and Sprat Bays) between February 6–12, 2016. Four traps were assigned to each site (Fig 1A–1C); in each site, one trap was assigned to and deployed in either *H*. *stipulacea*, *S*. *filiforme*, *T*. *testudinum*, or sand habitats daily at 7 am (± 0.5 h). Each trap was hauled five times between February 6–12, for a total of five trap samplings over the duration of 24 h deployments.

To investigate fine-scale diel variation in juvenile assemblages across native and invasive seagrasses, 12 h trap deployments were conducted in Brewers Bay between February 17–20, 2016. Five traps each were allocated to *H*. *stipulacea* and *S*. *filiforme* beds in Brewers Bay (Fig 1D) and deployed at least 10 m apart within the treatment habitat. Twice daily at 7 am and 7 pm (± 0.5 h), traps were collected and redeployed in both habitats. Each of the five traps in both treatment habitats was hauled for three consecutive nights and days between February 17–20, for a total of three nighttime trap samplings and three daytime trap samplings over the duration of 12 h deployments.

In the field, each trapped fish was identified to either species or family, its total length was measured (to the nearest mm), and it was photographed before being released. Fish that could not be identified at least to the taxonomic level of family in the field or from photographs were excluded from all analyses. Fish that exceeded the length at maturity [43–49] for their species were excluded from all analyses. When an individual could be identified only to family, the smallest length at maturity for all species in its family was used to provide the most conservative cutoff (Table 1). All families were categorized as either herbivores, diurnal carnivores or nocturnal carnivores [17, 50–52].

**Table 1:** Lengths at maturity for species identified in 24 and 12 h deployments.

Family	Scientific Name	L_m_ (cm)
Acanthuridae	*Acanthurus bahianus*	**15.5**
Carangidae	*Caranx latus*	**37**
Gerreidae	*Gerres cinereus*	**20**
Haemulidae	*Haemulon aurolineatum*	**14**
	*Haemulon flavolineatum*	16
	*Haemulon sciurus*	18.5
Holocentridae	*Holocentrus adscensionis*	14.6
	*Holocentrus rufus*	**13.5**
Lutjanidae	*Lutjanus griseus*	32
	*Lutjanus synagris*	23.8
	*Ocyurus chrysurus*	**22.5**
Mullidae	*Pseudopeneus maculatus*	18
	*Mulloidichthys martinicus*	**17**
Labridae	*Sparisoma aurofrenatum*	**17.5**

L_m_ = length of maturity, defined as the total length at which 50% of the population of that species becomes mature for the first time [43]. The smallest L_m_ (in bold) in each family was applied as the cutoff length for individuals that were identified only to family.

### Statistical analysis

Pielou’s evenness indices (J) and Simpson’s diversity indices (D) were calculated to compare family diversity between treatment habitats (*H*. *stipulacea*, *S*. *filiforme*, *T*. *testudinum*, and sand). For a more representative sample size, catch data from 24 h trap deployments were pooled across sites and days, and catch data from 12 h trap deployments were pooled across traps and days. Simpson’s D is defined in the present study as one minus the probability of randomly picking two individuals from the population that are the same species:

$$D=1-\sum_{i=1}^{S}p_{i}^{2}$$

where S is the number of species in a sample or population, and p_*i*_ is the fraction of a sample of individuals belonging to species *i*. This index was chosen because it is one of the simplest calculations of species diversity, and it is appropriate for aquatic systems [53]. Indices of family diversity are only nebulous descriptions of communities, but they can provide practical summaries of community structure when implemented as part of a more detailed analysis [54].

Non-metric multidimensional scaling (NMDS) biplots, based on the Bray-Curtis similarity measure, were generated to discern differences in juvenile assemblages across treatment habitats in each trap deployment period. Total abundance data were converted to relative abundances, and traps with only one fish were excluded when the family of that fish was unique. Ellipses, generated from the standard error of the weighted average of the scores in each treatment habitat, were superimposed on each biplot to visualize the dispersion of each treatment habitat group. A permutational analysis of variance (PERMANOVA) was conducted to test the multivariate response of fish family relative abundance to treatment habitats. When PERMANOVAs were significant (p < 0.05), a similarity percentage analysis (SIMPER) was performed to evaluate the contribution of each family to the difference between treatment habitats.

Univariate analyses were then used to explore differences in total abundance of juveniles across treatments and strata. Two-way analyses of variance (ANOVA) were conducted on the mean total abundance of juvenile fish (identified at least to family) across treatment habitats and sites in 24 h deployments and across treatment habitats and soak types (day vs. night) in 12 h deployments. To meet assumptions of homoscedasticity and equal variance, all data were square-root transformed prior to conducting ANOVAs.

Total abundance data were then separated by guild, and a variety of analyses were used to explore responses of each guild to treatment habitats and strata. For carnivores in 24 h deployments, data were square-root transformed, and a two-way ANOVA was used to test for a significant response of mean carnivore abundance to treatment habitats and sites. The data for each guild in 12 h deployments were zero-inflated. Generalized linear models (GLMs) were therefore used to test for significant responses in mean abundance of nocturnal carnivores, diurnal carnivores, and herbivores to treatment habitats and soak types. GLMs with a Poisson distribution were rejected after examination of the ratio of the residual deviance to the degrees of freedom suggested overdispersion. Alternatively, GLMs with negative binomial error structures and log-link functions were used. Starting at the null model, model fit was assessed incrementally by adding a predictor and conducting a *X*^2^ goodness of fit test. The best model was determined when, compared to a smaller model, the null hypothesis that the model with fewer predictors is adequate was rejected at the p < 0.05 level. For each guild, the predictor from the best fit model was determined to have a significant effect on the magnitude of the response at p < 0.05, and estimates (describing the response in *S*. *filiforme* relative to *H*. *stipulacea* for the seagrass predictor and describing the response in night relative to day for the soak type predictor) were used to assess the direction of the response between levels of the predictor. All statistical analyses were performed in R [55].

## Results

### Habitat characteristics

Monotypic stands of *H*. *stipulacea*, *S*. *filiforme*, and *T*. *testudinum* had average percent cover of 95.3%, 87.0% and 94.9% and average canopy heights were 6.13 cm, 15.05 cm, and 17.67 cm, respectively (Table 2). Both native species had taller canopies than *H*. *stipulacea*.

**Table 2:** Average percent cover and canopy height of seagrass patches used as trap deployment locations.

Seagrass	Site	Ave. Percent Cover ± St. Dev	Ave. Canopy Height ± St. Dev (cm)
***H*. *stipulacea***		**95.3 ± 7.95**	**6.13 ± 2.64**
	Brewers Bay	100 **±** 0.00	7.8 **±** 2.49
	Frenchman Bay	100 **±** 0.00	7.2 **±** 1.92
	Lindbergh Bay	83 **±** 2.74	5.58 **±** 2.09
	Sprat Bay	100 ± 0.00	2.5 **±** 0.5
***S*. *filiforme***		**87 ± 13.51**	**15.05 ± 4.33**
	Brewers Bay	100 **±** 0.00	14.6 **±** 3.36
	Frenchman Bay	94 ± 4.18	13 **±** 4.47
	Lindbergh Bay	79 **±** 6.52	19.3 **±** 4.02
	Sprat Bay	75 **±** 16.58	13.3 **±** 3.13
***T*. *testudinum***		**94.9 ± 3.90**	**17.67 ± 8.19**
	Frenchman Bay	99 **±** 2.24	15 **±** 3.54
	Lindbergh Bay	92 **±** 2.74	25.1 **±** 10.42
	Sprat Bay	93.8 **±** 2.77	12.9 **±** 2.75

Bold values indicate averages across sites.

### Juvenile assemblages

During 24 h deployments, a total of 117 fish were caught. Seven fish were not identified to the family level, and 27 fish were unable to be confirmed as juveniles. All analyses of 24 h deployments were completed on the remaining 83 juveniles. During 12 h deployments, a total of 188 fish were caught. Five fish were not identified to the family level, and 14 fish were unable to be confirmed as juveniles. All analyses of 12 h deployments were completed on the remaining 169 juveniles.

#### Diversity

The juvenile fish assemblage associated with *H*. *stipulacea* was consistently less diverse than that of native seagrasses. During 24 h deployments, *H*. *stipulacea* was associated with greater family richness but lower family diversity (n = 3 families, D = 0.21) than sand habitats (n = 2 families, D = 0.50); the native *T*. *testudinum* was associated with the greatest family richness and diversity (n = 5 families, D = 0.77) during this 24 h deployments. During 12 h deployments, *H*. *stipulacea* supported the lowest overall family richness and diversity during both nighttime (n = 3 families, D = 0.50) and daytime (n = 5 families, D = 0.61) soaks. Juvenile family richness and diversity were consistently greater in native *S*. *filiforme* during nighttime (n = 6 families, D = 0.67) and daytime (n = 5 families, D = 0.71) soaks (Table 3).

**Table 3:** Total catch of juvenile fish in 24 and 12 h trap deployments.

Trap Deployment Period and Site:		24 h: Frenchman, Lindbergh, and Sprat Bay				12 h: Brewers Bay			
						Soak Period: Day		Soak Period: Night	
Habitat:		*H*. *stipulacea*	*S*. *filiforme*	*T*. *testudinum*	*Sand*	*H*. *stipulacea*	*S*. *filiforme*	*H*. *stipulacea*	*S*. *filiforme*
**Nocturnal Carnivores:**	**Lutjanidae**	**38**	**8**	**4**	**4**	**9**	**10**	**19**	**13**
	**Haemulidae**	**4**	**5**	**3**	**4**	**11**	**6**	**29**	**23**
	**Holocentridae**	**1**	**1**	**3**	**0**	**0**	**0**	**0**	**0**
	**Sciaenidae**	**0**	**0**	**1**	**0**	**0**	**0**	**0**	**0**
	**Gerreidae**	**0**	**0**	**0**	**0**	**1**	**0**	**0**	**0**
**Diurnal Carnivores:**	**Mullidae**	**0**	**2**	**0**	**0**	**1**	**22**	**1**	**2**
	**Carangidae**	**0**	**0**	**0**	**0**	**0**	**1**	**0**	**1**
**Herbivores:**	**Acanthuridae**	**0**	**0**	**0**	**0**	**1**	**3**	**0**	**10**
	**Labridae**	**0**	**0**	**5**	**0**	**0**	**6**	**0**	**0**
**Total:**		**43**	**16**	**16**	**8**	**23**	**48**	**49**	**49**
**Family Richness:**		**3**	**4**	**5**	**2**	**5**	**6**	**3**	**5**
**Pielou’s Index of Evenness (J):**		**0.38**	**0.82**	**0.93**	**1**	**0.7**	**0.81**	**0.69**	**0.77**
**Simpson’s Index of Diversity (D):**		**0.21**	**0.63**	**0.77**	**0.5**	**0.61**	**0.71**	**0.5**	**0.67**

#### Community structure

**Fig 2:**
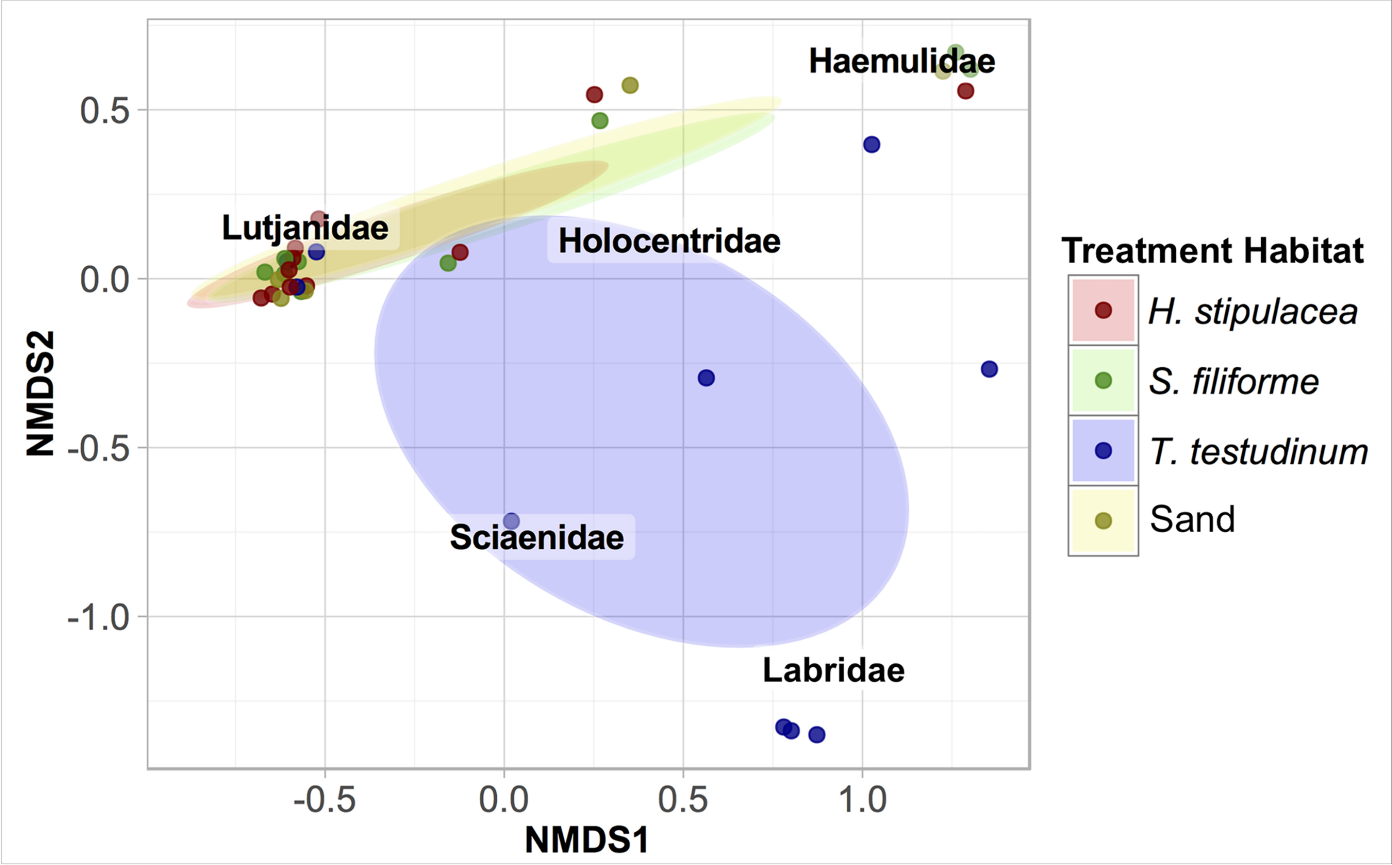
NMDS of each family’s relative abundance across benthic habitats during 24 h deployments. Points represent individual traps, and 95% confidence ellipses indicate sampling distributions for each habitat. Data were pooled across site.

During 24 h trap deployments, the juvenile fish communities associated with *H*. *stipulacea*, *S*. *filiforme*, and sand were relatively similar to each other but distinct from *T*. *testudinum* (Fig 2). The comparable communities of *H*. *stipulacea*, *S*. *filiforme*, and sand were driven by a considerable relative abundance of Lutjanidae and Haemulidae in each habitat. Communities in *T*. *testudinum* were uniquely shaped by a high relative abundance of Labridae (n = 5) and the presence of Sciaenidae (n = 1), as these families were absent from all other treatment habitats (Table 3). Overall, community structure differed significantly between *H*. *stipulacea*, *S*. *filiforme*, *T*. *testudinum*, and sand habitats (PERMANOVA F_3, 31_ = 2.1741, p = 0.014). A large relative abundance of juvenile Lutjanidae in *H*. *stipulacea* drove community structure differences between *H*. *stipulacea* and *T*. *testudinum* (SIMPER: 29.8% contribution to dissimilarity), *H*. *stipulacea* and *S*. *filiforme* (SIMPER: 18.7% contribution to dissimilarity), and *H*. *stipulacea* and sand (SIMPER: 17.8% contribution to dissimilarity). A large relative abundance of Labridae, a family unique to *T*. *testudinum*, drove further community structure differences between the native seagrass and *H*. *stipulacea* (SIMPER: 18.3% contribution to dissimilarity).

During 12 h trap deployments, *H*. *stipulacea* and *S*. *filiforme* supported similar nighttime communities but distinct daytime communities. Juvenile Lutjanidae and Haemulidae comprised the majority of the nighttime catch in *H*. *stipulacea* and *S*. *filiforme* as well as the daytime catch in *H*. *stipulacea*. The daytime catch in *S*. *filiforme* was dominated by Mullidae (n = 22), which outnumbered the combined catch of Haemulidae and Lutjanidae (n = 16, Table 3) during the daytime in *S*. *filiforme*. The daytime catch from *S*. *filiforme* also included herbivores (n = 3 Acanthuridae, n = 6 Labridae).

**Fig 3:**
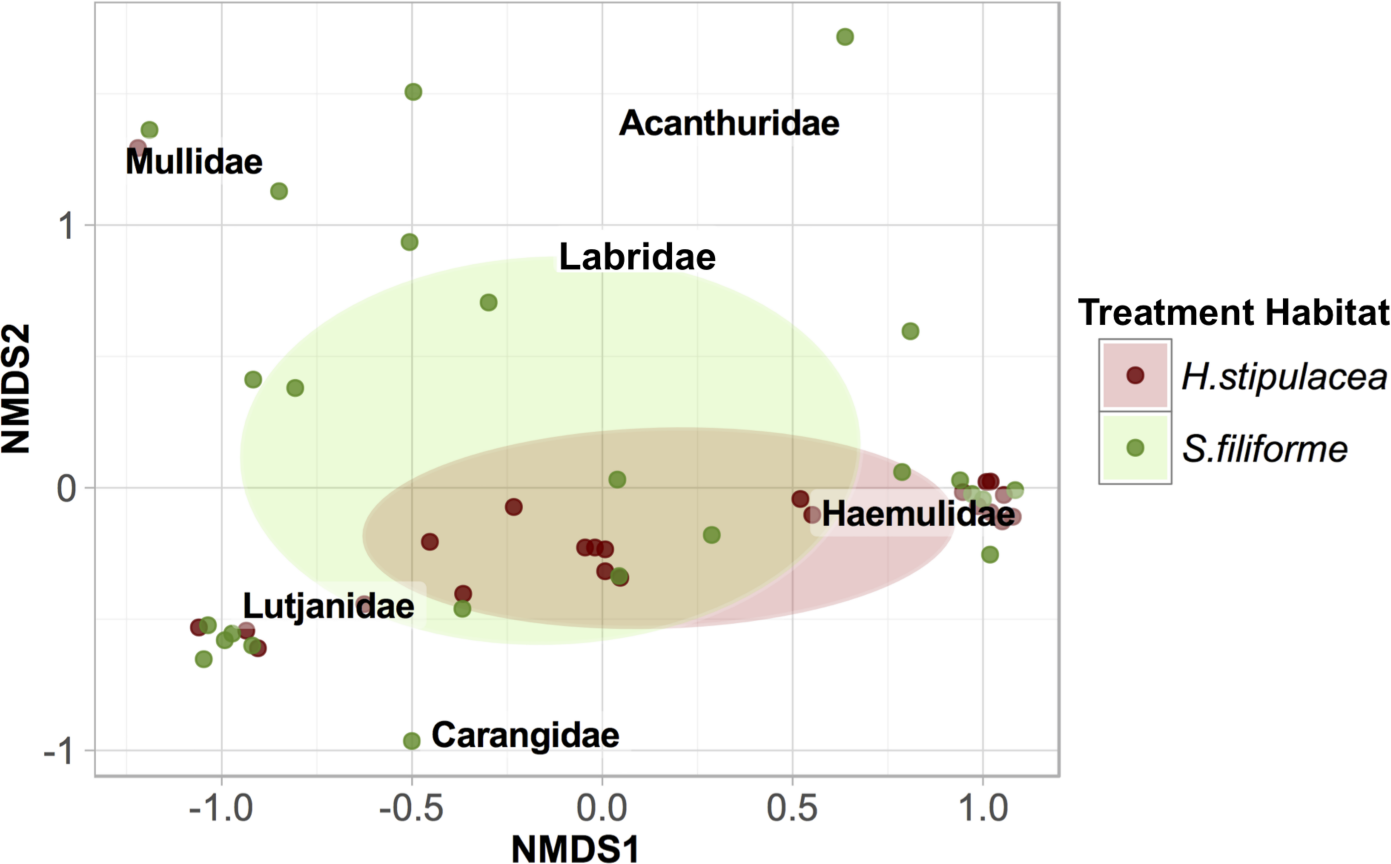
NMDS of juvenile fish family relative abundance during 12 h deployments. Points represent individual traps, and 95% confidence ellipses indicate sampling distributions for each seagrass. Data were pooled across soak types.

Nocturnal carnivores, diurnal carnivores, and herbivores were associated with *S*. *filiforme*, while the assemblage associated with *H*. *stipulacea* was comprised almost entirely of nocturnal carnivores. Despite greater family richness associated with *S*. *filiforme*, the community structure associated with *S*. *filiforme* overlapped with *H*. *stipulacea* (Fig 3) and community structure did not differ significantly between the two seagrass habitats (PERMANOVA: F1, 46 = 2.400, p = 0.070). The communities in each seagrass were pooled over soak types (daytime and nighttime), and similar nighttime abundances of Lutjanidae and Haemulidae drove statistically indistinguishable differences in community structure across seagrass habitats in 12 h deployments.

#### Abundance

**Fig 4:**
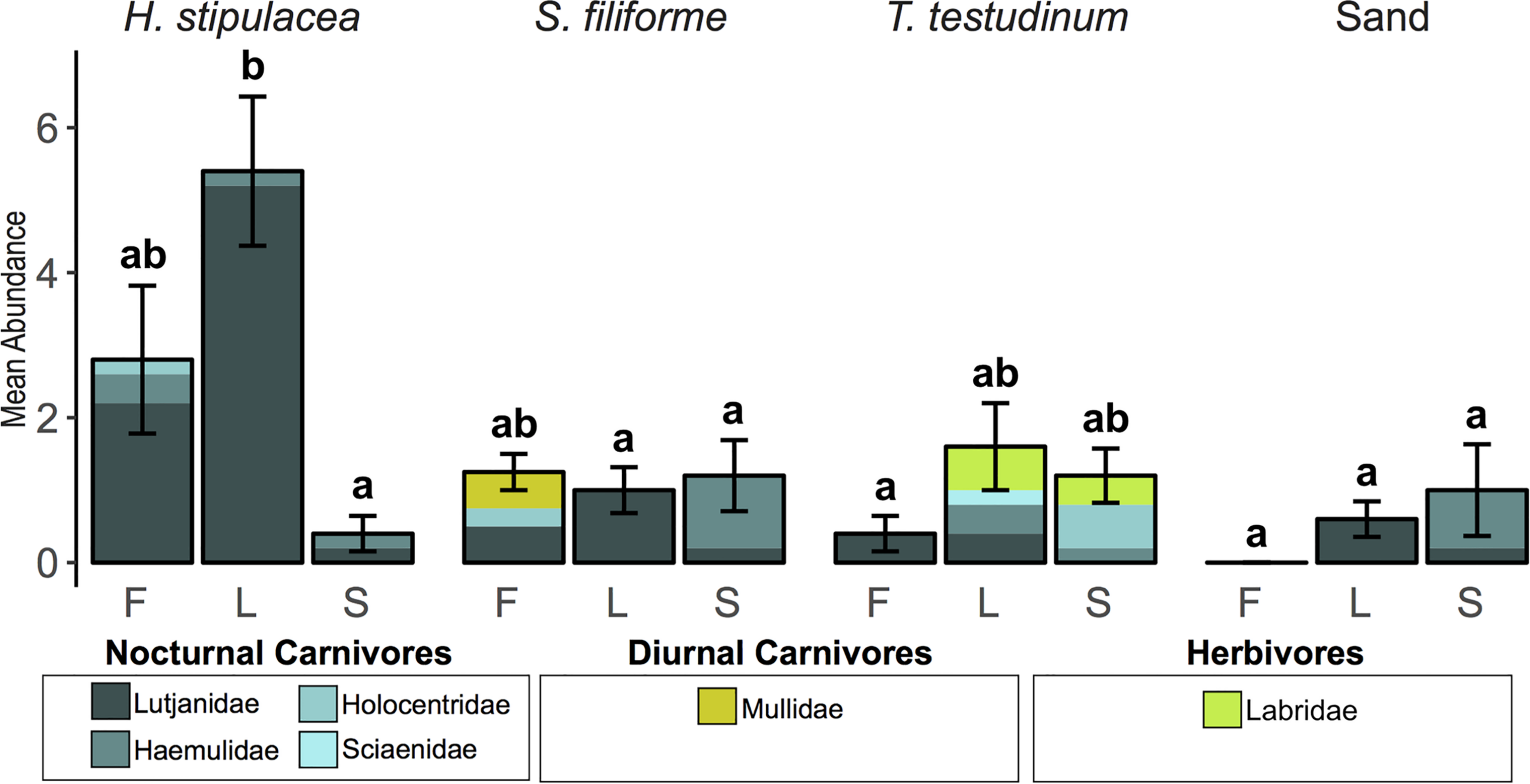
Mean juvenile fish abundance (± SEM) in each trap during 24 h trap deployments. Separated by treatment habitat (*H*. *stipulacea*, *S*. *filiforme*, *T*. *testudinum*, and sand) and site (F = Frenchman Bay, L = Lindbergh Bay, and S = Sprat Bay). The contribution of each family to the mean abundance is indicated by stacked colored bars. Letters from a Tukey HSD test indicate significant differences across sites and habitats.

Across 24 h trap deployments, average juvenile fish abundance differed significantly across habitats (p = 0.005), and sites (p = 0.029), with a significant interaction (p = 0.007) (Table 4A) (Fig 4). Juveniles were significantly more abundant in *H*. *stipulacea* than in sand habitats (Tukey test p = 0.002) and in Lindbergh Bay than in Sprat Bay (Tukey test p = 0.039). A significant interaction was due to a greater juvenile abundance in Lindbergh Bay *H*. *stipulacea* compared to Sprat Bay *H*. *stipulacea* (Tukey test p = 0.0013) (Fig 4).

**Table 4:** Two-way analyses of variance (ANOVAs).

	Factor	**df**	**F**	**p**
**a. 24 h Deployments: Total Catch**	Treatment Habitat	3	4.949	0.005 *
	Site	2	3.822	0.029 *
	Treatment Habitat x Site	6	3.452	0.007 *
**b. 24 h Deployments: Carnivores**	Treatment Habitat	3	5.726	0.002 *
	Site	2	3.374	0.043 *
	Treatment Habitat x Site	6	2.924	0.017 *
**c. 12 h Deployments: Total Catch**	Habitat	1	0.71	0.403
	Soak Type	1	3.673	0.06
	Treatment Habitat x Soak Type	1	0.943	0.336

Carnivores (both nocturnal and diurnal) comprised 94% of the total catch of 83 juvenile fish in 24 h deployments. The nocturnal carnivore family Lutjanidae were the most abundant family (n = 54); other carnivores included nocturnal families Haemulidae, Holocentridae, and Sciaenidae (n = 18) as well as the diurnal family Mullidae (n = 2) (Table 3). Five herbivores (Labridae) represented the remaining 6% of total fish abundance, and all Labridae were caught in *T*. *testudinum* beds in either Sprat Bay (n = 2) or Lindbergh Bay (n = 3).

Because carnivores comprised the majority of the total catch, results from two-way ANOVAs on carnivore abundance were similar to two-way ANOVAs on total abundance. Carnivore abundance differed significantly across habitats (p = 0.002) and sites (p = 0.043), with an interaction (p = 0.017) (Table 4B). Carnivores were more abundant in *H*. *stipulacea* than in *T*. *testudinum* (Tukey test p = 0.008) and sand habitats (Tukey test p = 0.003). Carnivores were also significantly more abundant in Lindbergh Bay than in Sprat Bay (Tukey test p = 0.038). A significant interaction was driven by a greater abundance of carnivores in Lindbergh Bay *H*. *stipulacea* than in Sprat Bay *H*. *stipulacea* (Tukey test p = 0.044).

**Fig 5:**
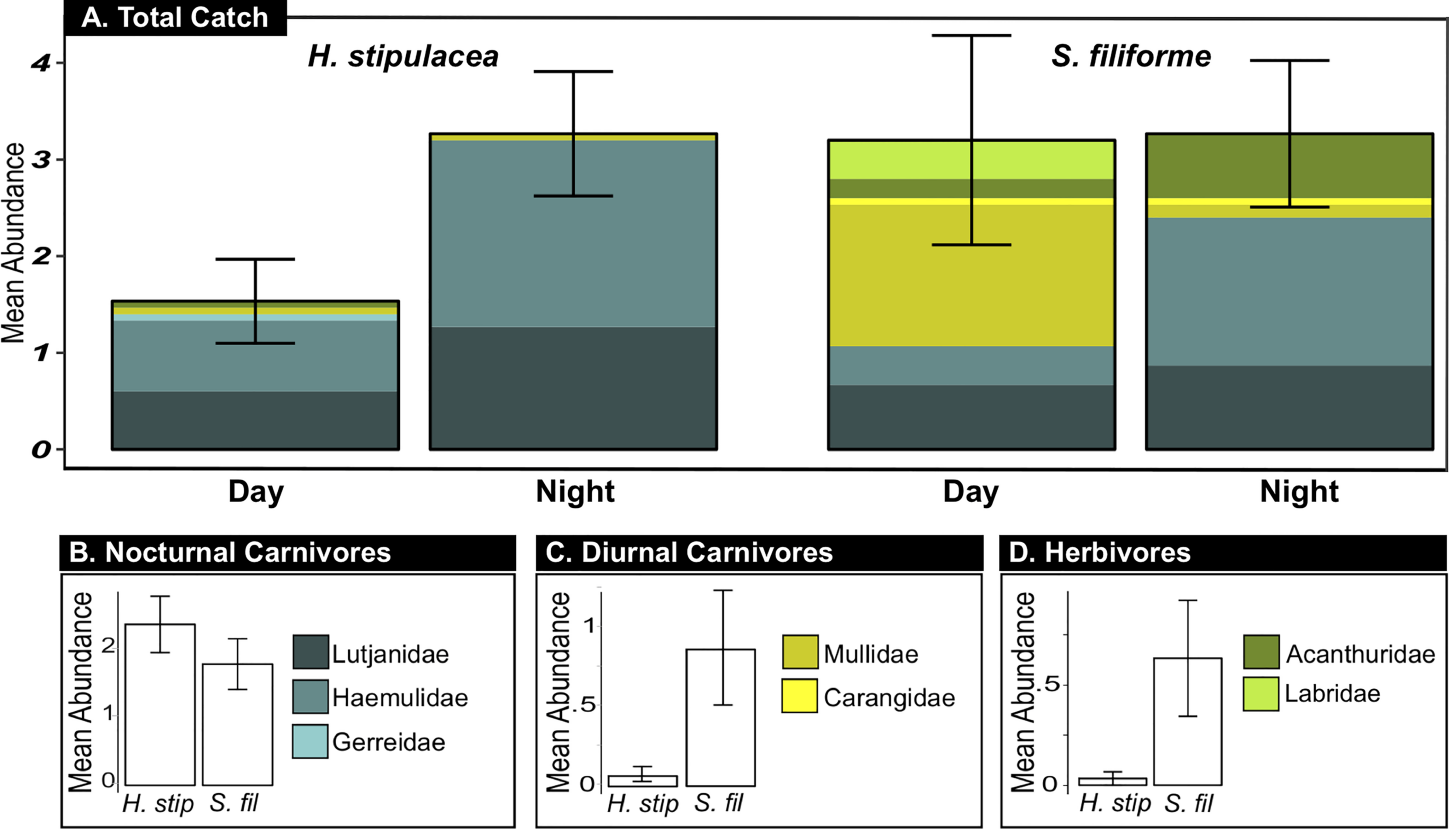
Mean juvenile fish abundance in 12 h trap deployments. (A) Mean abundance of the total catch (**±** SEM), separated by treatment habitat and soak type. The contribution of each family to the mean abundance is indicated by stacked colored bars. For each guild, a legend with colors corresponding to each family and plots of the mean abundance (**± SEM)** across seagrass habitats are given for (B) nocturnal carnivores, (C) diurnal carnivores, and (D) herbivores.

Across 12 h trap deployment periods in Brewers Bay, mean juvenile fish abundance did not significantly differ between seagrass beds (p = 0.403) or soak type (night vs. day) (p = 0.060) (Table 4) (Fig 5A). Significant differences emerged when analyses were conducted on separate guilds.

GLMs revealed that nocturnal carnivore abundance differed across soak types and diurnal carnivore and herbivore abundance differed across seagrass habitats. Nocturnal carnivores were more abundant at night, but their abundance did not differ significantly between *H*. *stipulacea* and *S*. *filiforme* habitats (Table 5) (Fig 5B). Haemulidae comprised the majority of the catch in both seagrasses at night (n = 52 in *H*. *stipulacea*, n = 32 in *S*. *filiforme*), but much fewer were caught during the day in either seagrass (n = 17 in *H*. *stipulacea*, n = 19 in *S*. *filiforme*) (Fig 5A). Diurnal carnivores and herbivores were significantly more abundant in *S*. *filiforme* than in *H*. *stipulacea* (Table 5) (Fig 5C and 5D). While diurnal carnivores were abundant in *S*. *filiforme* (n = 24 Mullidae, n = 2 Carangidae), only two (both Mullidae) were caught in *H*. *stipulacea* over the duration of the experiment (Fig 5A). Similarly, herbivores were abundant in *S*. *filiforme* (n = 6 Labridae, n = 13 Acanthuridae), but only one Acanthuridae was trapped in *H*. *stipulacea* over the duration of the experiment (Fig 5A).

**Table 5:** Negative binomial generalized linear model results for each guild during 12 h deployments.

	Nocturnal Carnivores			Diurnal Carnivores			Herbivores		
	Est. ± SE	z	p	Est. ± SE	z	p	Est. ± SE	z	p
Intercept:	0.34 ± 0.23	1.47	0.14	-2.7± 0.80	-3.38	**0.0007**	-3.4 ± 1.09	-3.12	**0.00179**
Seagrass (HS–SF):	-0.28 ± 0.25	-1.12	0.26	2.56 ± 0.91	2.82	**0.0048**	2.94 ± 1.19	2.47	**0.01367**
Soak Type (Day–Night):	0.82 ± 0.26	3.18	**0.001**						
Res. Dev. (Null Dev.):	65.985 (77.734)			27.711 (37.511)			21.496 (30.028)		

HS = *H*. *stipulacea*. SF = *S*. *filiforme*. Res. Dev. = residual deviance. Null Dev. = null deviance.

## Discussion

This study is the first to characterize juvenile fish assemblages in St. Thomas seagrass habitats, and findings suggest reduced family diversity and altered assemblages of juvenile fish in habitats composed of the invasive seagrass *H*. *stipulacea*. During 24 h deployments, nocturnal carnivore abundance (primarily Lutjanidae) was significantly greater in *H*. *stipulacea* habitats than in any other seagrasses. During 12 h deployments, however, nocturnal carnivore abundance (comprising Lutjanidae and Haemulidae) was indistinguishable across seagrasses. Diurnal carnivores were scarce in *H*. *stipulacea* habitats during both deployment periods, while the diurnal carnivore family Mullidae constituted the majority of the catch from daytime soaks in *S*. *filiforme* habitats. Herbivores were also scarce in *H*. *stipulacea* during both deployment periods. The few herbivores caught during 24 h deployments (all Labridae) were trapped only in *T*. *testudinum*, and herbivore families Labridae and Acanthuridae were trapped in *S*. *filiforme* during both daytime and nighttime soaks. Overall, the invasive may be preferred habitat for nocturnal carnivores but is likely suboptimal for diurnal carnivores and herbivores. Continued invasion of *H*. *stipulacea* and subsequent loss of native seagrasses may thus diminish preferred habitats for some juvenile fish.

Comparison between this study and previous reports is important yet challenging due to differences in sampling technique, habitats, seasons, geographic localities, and environmental conditions [42]. The catch data from this study reflects a relatively low diversity compared to elsewhere in the Caribbean, but this could be partly explained by an exclusive focus on the juvenile life stage. Compared to studies that report catch-data inclusive of all life stages, a focus on juveniles in the current study led to the omission of families when individuals exceeded L_m_; for example, adults belonging to the family Tetraodontidae were caught on multiple occasions during 24 h deployments. Juvenile fish diversity may also be reduced from historical levels [56] due to overfishing of reproductive adults, and therefore diminished larval supply, of many commercially important species in St. Thomas waters [31].

The identity of habitats surrounding seagrass beds is an important driver of seagrass-associated juvenile assemblages. In both the present study and previous reports from Dominica, mangrove habitats were absent surrounding seagrass study sites [22]. In contrast, studies from Curacao and Guadeloupe were conducted near large red mangrove forests in shallow (<2 m) seagrass habitats [3, 57]. The catch of Lutjanidae from both the present work and Dominica was dominated by small-bodied species, namely *Lutjanus synagris*, while the catch from both Curacao and Guadeloupe was dominated by juveniles of large-bodied, mangrove-associated species such as *Ocyurus chrysurus*, L. *griseus*, and *L*. *apodus* [3, 57, 58]. Though less abundant, herbivore assemblages in the present study were also more similar to the catch from Dominica. Labridae were absent from seagrasses adjacent to mangroves in Guadeloupe, and *Acanthurus bahianus* was notably absent from seagrasses adjacent to mangroves in both Curacao and Guadeloupe [3, 22, 57]. Moreover, species such as *Haemulon flavolineatum* (family Haemulidae) and *Pseudopeneus maculatus* (family Mullidae) were relatively abundant in the current study, but absent from mangrove-associated seagrass habitats in Guadeloupe [3]. Species and life-stage specific habitat preference can explain discrepancies in juvenile assemblages across these different habitats. Some reef-associated species (e.g. *A*. *bahianus*) may remain close to the reef and avoid seagrass-mangrove habitats, some (e.g. *O*. *chrysurus*) may initially inhabit sheltered seagrass and mangrove habitats but gradually migrate to seagrass habitats adjacent to reef as they grow, and others (e.g. *L*. *griseus* and *L*. *apodus*) may remain in seagrass-mangrove habitats throughout the majority of their juvenile life-stage [3, 57].

Physical characteristics, particularly canopy height, determine what protection and food resources a seagrass habitat may provide [59]. Canopy heights reported from this study are similar to those previously reported from Dominica (20 cm for *S*. *filiforme*, 18 cm for *T*. *testudinum*, and 4 cm for *H*. *stipulacea*) [24]. Varying canopy heights among the three seagrass species may partly explain why fewer families were present in *H*. *stipulacea* than in native seagrasses. In the present study, the native seagrasses *T*. *testudinum* and *S*. *filiforme* had the tallest canopies and also supported the most diverse assemblages. The patterns of juvenile fish diversity reported in the present work corroborate previous studies that linked tall canopies and greater fish diversity [59]. Moreover, the canopy height of *H*. *stipulacea* was recently attributed to reduced diversity of associated fish assemblages in Bonaire [33]. Juveniles may be deterred from using *H*. *stipulacea* habitats if the invasive species’ short canopy provides less protection, increases predation risk, and reduces probability of survival [60].

Contrary to the association between seagrass canopy height and increased juvenile fish diversity, diversity indices indicate that sand supported a more diverse assemblage than *H*. *stipulacea*. The Simpson’s D for sand habitats was inflated, however, as a result of a small catch and incidentally perfect species evenness. In general, juvenile fish prefer vegetated habitats over unvegetated habitats [61], and this is in line with the present findings of greater abundance in *H*. *stipulacea* and greater diversity in native seagrasses, compared to sand. *H*. *stipulacea* opportunistically expands into unvegetated substrates (S1 Fig) and has driven elimination of sand patches and reef halos in Dominica [62]. Though not preferred by juvenile fish, sand habitats are important for schooling fish and large mobile individuals [61]. While the invasive seagrass, by its vegetative nature, may support a greater abundance of juvenile fish, species that depend on sand habitats may suffer habitat loss due to expansion of *H*. *stipulacea*.

Nocturnal carnivores were more abundant in *H*. *stipulacea* than in native seagrasses during 24 h deployments, implying that the juveniles of some species belonging to this group may prefer *H*. *stipulacea*. Preference for *H*. *stipulacea* may be due to an abundance of preferred food resources associated with the invasive seagrass. In Dominica, amphipod epibionts were more abundant on *H*. *stipulacea* than in *S*. *filiforme* [22], and amphipods are preferred food items for juvenile *L*. *synagris* [63], the most abundant nocturnal carnivore in 24 h deployments. It has been suggested that epibiont abundance in *H*. *stipulacea* is a result of its densely matted structure [22], and *H*. *stipulacea* treatment habitats in the present study were composed of dense mats of the invasive seagrass.

Contradictory to results from 24 h deployments, nocturnal carnivore abundance in 12 h trap deployments did not differ across treatment habitats. This inconsistency may be a product of limited temporal extent and sample size, but other explanations are proposed. Conflicting results could be due to dissimilar abundances of Lutjanidae and Haemulidae across trap deployment periods, as species belonging to these families exhibit a diverse range of habitat preferences [2]. The catch from 24 h deployments was dominated by *L*. *synagris*, but *H*. *aurolineatum*, *H*. *flavolineatum*, and *L*. *synagris* were similarly abundant in 12 h deployments. *H*. *aurolineatum*, which were almost exclusively caught in *S*. *filiforme* in 12 h deployments, may prefer the native seagrass. Previous gut content analyses revealed that juvenile Haemulidae consume mainly tanaid prey [64], while juvenile *L*. *synagris* prefer amphipod prey [63]. Amphipods were the only crustaceans previously quantified in *H*. *stipulacea* [22], however, so it remains unknown whether tanaid abundance differs between *H*. *stipulacea* and *S*. *filiforme*. *H*. *aurolineatum* may also forage in *S*. *filiforme* as a result of competitive exclusion from *H*. *stipulacea* habitats; populations that migrate daily from large coral reefs to smaller seagrass habitats likely face heightened competition for food and space [64].

The orientation of *H*. *stipulacea* and *S*. *filiforme* in Brewers Bay may also explain inconsistent findings in nocturnal carnivore abundance across 24 and 12 h deployment periods. Juvenile Lutjanidae and Haemulidae are highly mobile while foraging and thus may migrate haphazardly between seagrass patches [5]. The habitat configuration in Brewers Bay may facilitate such migration; while treatment habitats in the 24 h deployment sites were separated by sand, the border between the two treatment habitats in Brewers Bay was comprised of a mixed stand of *S*. *filiforme* and *H*. *stipulacea*. A sheltered corridor between the two treatment habitats (provided by continuous vegetation) may permit juveniles to use the Brewers Bay seascape as a spatial continuum instead of discrete patches. Haphazard use of *H*. *stipulacea* and *S*. *filiforme* habitats has been discussed previously [22], and facilitation of this indiscriminate behavior (by a vegetated corridor) may have led to indistinguishable abundances across Brewers Bay treatment habitats.

Nocturnal carnivores were more abundant in nighttime soaks than in daytime soaks, reflecting this group’s daily transition between habitats. Previous studies suggest that Lutjanidae and Haemulidae use seagrasses as a foraging habitat at night and nearby coral reefs as sheltering habitats during the day [64]. Diel variation in habitat use is a way to take advantage of available resources through balancing high growth rates (abundant nighttime food resources provided by seagrass beds) with low predation-induced mortality (ample daytime shelter provided by coral reefs) [65]. The juveniles abundant in these seagrasses at night likely migrated to nearby coral reefs during daylight hours.

Unlike nocturnal carnivores, juvenile diurnal carnivores belonging to the Mullidae family may avoid habitats composed of *H*. *stipulacea*. Diurnal carnivores were most abundant in Brewers Bay *S*. *filiforme* habitats during 12 h deployments, and *P*. *maculatus* was the most common species identified. *P*. *maculatus*, like most Mullidae, are benthic carnivores that forage in and ingest sediments to consume their preferred food, and their specialized diets drive their requirement for sparsely vegetated foraging habitats [66, 67]. Habitat alterations resulting from the invasion of the marine alga *Caulerpa taxifolia* to the French Mediterranean coast were recently linked to restricted foraging of the striped red mullet *Mullus surmuletus* (family Mullidae). It was suggested that the invasion of *C*. *taxifolia* could increase intraspecific competition and restructure *M*. *surmuletus* populations [68]. *H*. *stipulacea* is structurally similar to the densely matted invasive macroalgae, and its displacement of *S*. *filiforme* in Dominica led to a loss of space under seagrass canopies and between seagrass shoots [24]. Similar to the response of *M*. *surmuletus* to invasion by *C*. *taxifolia*, heightened intraspecific competition among *P*. *maculatus* populations may result from invasion of *H*. *stipulacea* and loss of preferential foraging habitat.

Juvenile herbivores may prefer native seagrasses *T*. *testudinum* and *S*. *filiforme* habitats over *H*. *stipulacea*. Labridae was the most abundant family and shaped the unique community associated with *T*. *testudinum*. Three out of five herbivores caught in *T*. *testudinum* were *Sparisoma aurofrenatum*. The association between *S*. *aurofrenatum* and this seagrass corroborates a study from the Florida Keys, in which patch reefs and surrounding *T*. *testudinum* beds were identified to be the home range of *S*. *aurofrenatum* [69]. The absence of herbivores in *S*. *filiforme* during 24 h deployments, however, is inconsistent with previous findings that selective grazing on *S*. *filiforme* by *S*. *aurofrenatum* and other herbivores plays a role in zonation of *S*. *filiforme* and *T*. *testudinum* [70]. The absence of herbivores in *S*. *filiforme* during 24 h deployments is not too notable, however, considering that a total of only five juvenile herbivores were trapped during this period.

Proximity to reef in Brewers Bay may explain why herbivores were more abundant in 12 h deployments than in 24 h deployments. Herbivores tend to be more abundant in closer proximity to reefs [3], and seagrass patches in Brewers Bay were relatively close to a well-developed reef, unlike the treatment habitats in Frenchman, Lindbergh, and Sprat Bays. During daytime trap deployments, herbivores were absent from *H*. *stipulacea*, perhaps implying that food resources provided by the invasive are sub-optimal. If juveniles graze on seagrass blades, the nutritional content of *H*. *stipulacea* may be inferior to *S*. *filiforme*, as previous studies suggest that minor changes in nutritional content can sway herbivore preference for one seagrass over another [71]. If juveniles instead graze on epiphytic algae, the algal community of *H*. *stipulacea* may be inferior to *S*. *filiforme*. In the Mediterranean, leaves of *H*. *stipulacea* were found to support a poor variety of epiphytic flora and a distinct absence of encrusting coralline algae, compared to other seagrasses [34].

The catch of Acanthuridae during 12 h deployments was unexpected; the nighttime catch abundance of this diurnal group was double that of the daytime abundance. Fewer Acanthuridae during daytime soaks may be explained by the highly mobile behavior of this family; individuals been observed frequently entering and exiting traps during active daytime hours [41]. The nighttime catch of Acanthuridae in the present study was also inconsistent with previous observations that this family migrates from seagrass beds to nearby reefs at dusk to seek shelter [2]. It is possible that traps provided an incidental source of nighttime shelter and attracted more juvenile Acanthuridae than *S*. *filiforme* would otherwise harbor at night.

This study is one of only a few to characterize juvenile fish communities at multiple sites around the same island [42]. Uniform sampling permitted robust investigation of juvenile assemblages in native and invasive seagrasses across four sites. Patterns of nocturnal carnivore abundance across treatment habitats varied with site, with Lindbergh Bay *H*. *stipulacea* habitats supporting the greatest abundance. Though environmental quality data were not collected in the present study, an interaction between treatment habitat and site may be due to site-specific water temperature and salinity. Lindbergh Bay is subjected to high levels of terrestrial runoff due to urban development, and the bay receives inputs of thermohaline effluent from a nearby power plant [72]. Seagrass-associated juvenile fish assemblages in non-estuarine embayments can be influenced by salinity and temperature changes, driving increased density of a select few juvenile species but reduced density of most others [73]. Such patterns have only been linked to seasonal drops in salinity and temperature due to rainfall, however, and no reports could be found linking terrestrial runoff to altered juvenile communities in tropical seagrass ecosystems.

The present study is limited in sample size and temporal extent, compared to similar studies that carried out more than 20 trap pulls per seagrass habitat over multiple months [22]. Replication in the present study was limited because few traps were deployed in each treatment habitat (n = 1 and 5 habitat^-1^ site^-1^ in 24 and 12 h deployments, respectively), and trap samplings took place within a brief period (Feb 6–20, 2016). Because juvenile assemblages vary greatly with seasonal changes in water temperatures as well as wind speed and direction, a thorough characterization of juvenile assemblages requires repeated field surveys across seasons [74, 75]. Limited replication and temporal extent prevents broad conclusions from being drawn from the present study, and such limitations could be responsible for conflicting results in nocturnal carnivore abundance across trap deployment periods. Consistent differences in assemblage structure between native and invasive seagrasses identified in the present work, however, represent a potentially concerning consequence of the invasion of *H*. *stipulacea*.

### Future work and management implications

Despite the rapid takeover of *H*. *stipulacea* in many parts of the Caribbean, few studies explore potential trophic consequences of this invasion. Further trapping studies that address a broader temporal or geographic extent would be valuable to establish the degree of seasonal and cross-island variability in juvenile assemblages associated with *H*. *stipulacea*. Additionally, combining stable isotope and fatty acid analyses with trapping experiments would be instrumental in resolving uncertainties about species-specific foraging in *H*. *stipulacea;* previous work has demonstrated the use of these techniques in determining where fish are foraging and what they are consuming [9, 76]. Dietary analyses will be more informative once the nutritional content and algal communities of Caribbean *H*. *stipulacea* are characterized, similar to how the crustacean epibiota associated with the invasive have been described [22]. If future studies corroborate the results of the present work, the abundance, growth, and mortality of juvenile herbivores and diurnal carnivores should be monitored in bays where *H*. *stipulacea* is displacing preferable nursery habitat. Because large-scale habitat characteristics (on the scale of hundreds of meters) can play a considerable role in structuring reef fish populations [77], future research should also address the impacts to fish communities as a result of the homogenizing effect of *H*. *stipulacea*. Lastly, the expansion of *H*. *stipulacea* should be monitored closely, with particular regard to its ongoing spread to Caribbean islands, colonization following disturbance, and displacement of native seagrasses.

Special management priority should be given to seagrass habitats known to be key juvenile fish nurseries because alterations to these seascapes could disproportionately affect the surrounding habitats to which juveniles eventually recruit [1]. In Marine Protected Areas (MPAs) where *H*. *stipulacea* invasions have already occurred, such as the Virgin Islands National Park [23], special assessments should be made to determine the magnitude of threat that the invading seagrass poses to juvenile fish assemblages. Key juvenile habitats not presently within MPA boundaries should be identified using improved spatial prediction of juvenile fish species richness and abundance and prioritized in regards to MPA candidacy [74]. The present study demonstrates that *H*. *stipulacea* may be a suboptimal nursery habitat for herbivores and diurnal carnivores, and further monitoring should be carried out to identify species-specific patterns in juvenile use of habitats composed of the invasive seagrass. Functional redundancy of juvenile habitats is already reduced around the urbanized island of St. Thomas; intense coastal development has resulted in the loss of most mangrove habitats [78], another key juvenile fish habitat. In St. Thomas, therefore, native seagrasses may be the only remaining preferable habitats for some juvenile species, and displacement of these seagrasses by *H*. *stipulacea* may reduce survivorship if juveniles are obligated to seek suboptimal nursery habitat.

Efforts should be made to inhibit the spread of *H*. *stipulacea* to other Caribbean islands (e.g. Puerto Rico) and Florida through enforcement of proper recreational and commercial boating and anchoring practices. This will help minimize fragmentation and vegetative propagation, the principle means of *H*. *stipulacea* dispersal throughout the Caribbean [23]. Once *H*. *stipulacea* has invaded, a number of actions may help mitigate the negative impacts of its introduction. While some suggest that physical removal of the invasive will be ineffective [28], others have proposed dredging *H*. *stipulacea* beds and seeding areas with native seagrasses [79, 80]. One alternative to complete removal is selective extraction of dense mats of *H*. *stipulace*a, which may prevent sediment anoxia and liberate foraging habitat for benthic carnivores [27, 68]. Working to minimize the spread of *H*. *stipulacea* in tropical nearshore ecosystems will be vital to preserving landscape complexity, habitat quality, and ultimately biodiversity in these productive environments.

## Supporting information

S1 Fig *H*. *stipulacea* patch and nearby coral reef in perseverance bay, St. Thomas (Photo credit: L. K. Olinger). (TIF)
