# Supplementary figures and images for: Altered juvenile fish communities associated with invasive Halophila stipulacea seagrass habitats in the U.S. Virgin Islands
Source: PLoS One. 2017 Nov 21;12(11):e0188386. doi: 10.1371/journal.pone.0188386 (PMC5697852; doi:10.1371/journal.pone.0188386)

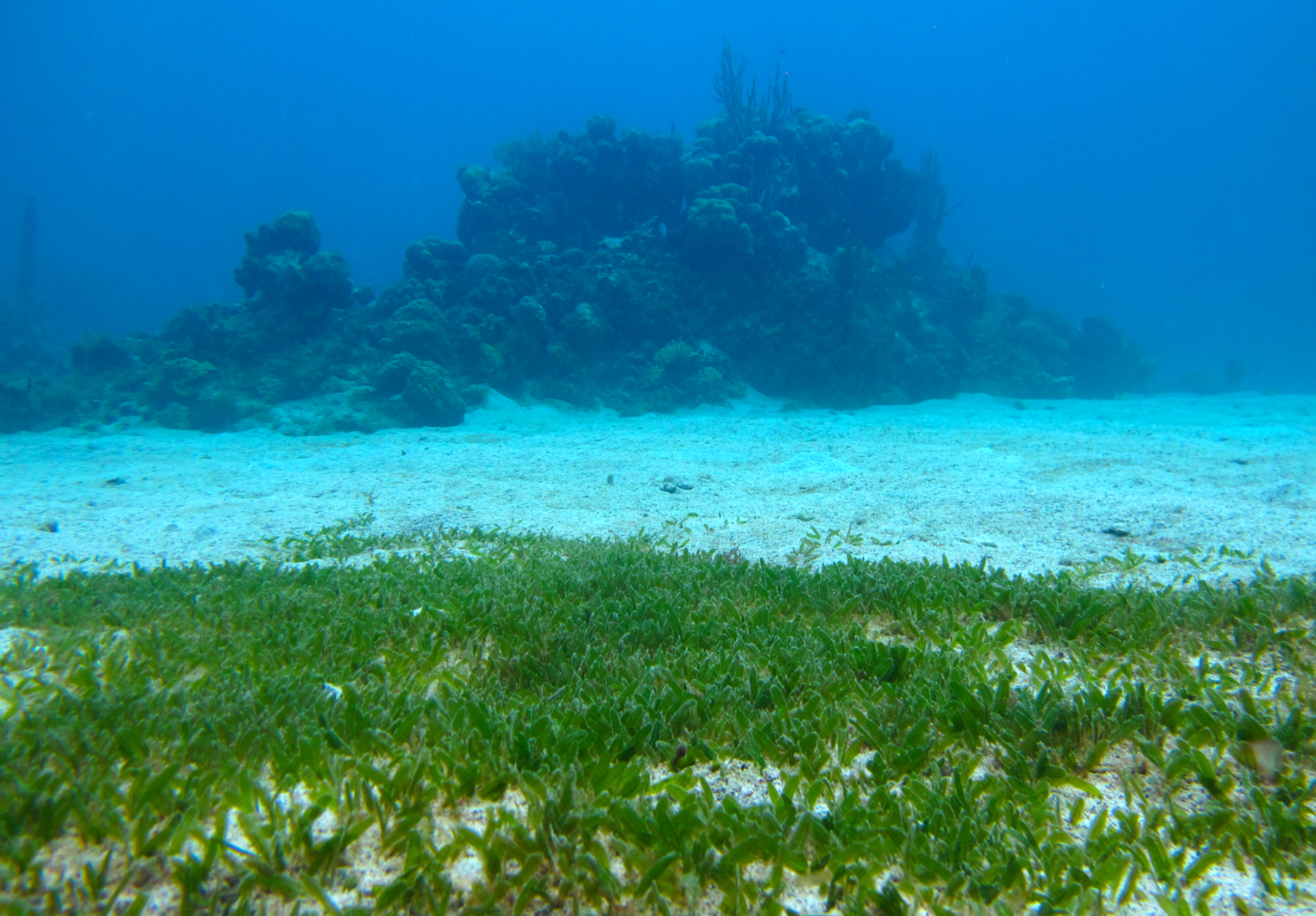

Supplement: S1 Fig — (TIF) [file pone.0188386.s001.tif]
